# Natriuretic Peptides Regulate Prostate Cells Inflammatory Behavior: Potential Novel Anticancer Agents for Prostate Cancer

**DOI:** 10.3390/biom11060794

**Published:** 2021-05-25

**Authors:** Letizia Mezzasoma, Vincenzo Nicola Talesa, Egidia Costanzi, Ilaria Bellezza

**Affiliations:** Department of Medicine and Surgery, University of Perugia, Polo Unico Sant’Andrea delle Fratte, P.le L. Severi, 1, 06132 Perugia, Italy; vincenzo.talesa@unipg.it (V.N.T.); egidia.costanzi@unipg.it (E.C.); ilaria.bellezza@unipg.it (I.B.)

**Keywords:** Atrial Natriuretic Peptide, B-Type Natriuretic Peptide, inflammation, tumor microenvironment, extracellular vesicles, inflammasome, IL-1β, caspase-1, ERK1/2 MAPK, P38-MAPK

## Abstract

Inflammation, by inducing a tumor-promoting microenvironment, is a hallmark for prostate cancer (PCa) progression. NOD-like receptor protein 3 (NLRP3)-inflammasome activation, interleukin-1β (IL-1β) secretion, and cancer cell-released extracellular vesicles (EVs) contribute to the establishment of tumor microenvironment. We have shown that PC3-derived EVs (PC3-EVs) activate inflammasome cascade in non-cancerous PNT2 cells. It is known that the endogenous biomolecules and Natriuretic Peptides (NPs), such as ANP and BNP, inhibit inflammasome activation in immune cells. Here we investigated whether ANP and BNP modify PCa inflammatory phenotype in vitro. By using PNT2, LNCaP, and PC3 cell lines, which model different PCa progression stages, we analyzed inflammasome activation and the related pathways by Western blot and IL-1β secretion by ELISA. We found that tumor progression is characterized by constitutive inflammasome activation, increased IL-1β secretion, and reduced endogenous NPs expression. The administration of exogenous ANP and BNP, via p38-MAPK or ERK1/2-MAPK, by inducing NLRP3 phosphorylation, counteract inflammasome activation and IL-1β maturation in PC3 and PC3-EVs-treated PNT2 cells, respectively. Our results demonstrate that NPs, by interfering with cell-specific signaling pathways, exert pleiotropic anti-inflammatory effects converging toward inflammasome phosphorylation and suggest that NPs can be included in a drug repurposing process for PCa.

## 1. Introduction

Prostate cancer (PCa) is the second most common cause of cancer and the sixth cause of cancer death among men worldwide [1]. Localized PCa is usually treated with surgery or radiation, whereas androgen deprivation therapy represents the best therapeutic option for metastatic tumors. Nevertheless, initial regression is frequently followed by a tumor relapse into a castration resistant phenotype for which no therapeutic treatment is presently available [2,3]. Alongside the well-established factors involved in PCa, chronic inflammation, caused by virus or microbial infections, represents a hallmark of PCa development and progression, as well as of the development of androgen independence [4,5].

By secreting cytokines, chemokines, and others soluble factors, inflammatory cells on one hand and cancer cells on the other, orchestrate the recruitment of a wide variety of immune cells and incessantly modify the surrounding tumor microenvironment. These events culminate in immunosuppression, cancer progression, and increased tumor aggressiveness [4].

Tumor microenvironment complexity is further enhanced by cancer-released extracellular vesicles (EVs), key mediators of cell–cell communications. EVs contain a cargo of molecules able to alter the behavior of microenvironment residing or infiltrating cancerous and non-cancerous cells, thus triggering a vicious circle, which drives cellular activities toward cancer progression and aggressiveness [6].

PCa secrete EVs able to modify tumor microenvironment [7]. We have demonstrated that EVs released from advanced-stage of PCa cells, by activating the inflammasome cascade and interleukin-1β (IL-1β) production, alter the immune/inflammatory response of microenvironment-residing cells in a tumor promoting fashion [8].

IL-1β is one of the most abundant and influential cytokine of tumor microenvironment [9,10]. Inflammation-associated enhancement of IL-1β exerts immunosuppressive effects in tumor microenvironment and supports tumor progression and metastatic processes in many different human malignancies [9,10,11,12,13]. High IL-1β levels have been found in tumors [14] and in sera [15] of patients with advanced PCa. As a result of the pleiotropic effect of IL-1β, molecules able to modulate its activities have been proposed as candidates for anti-tumor therapy approaches [10]. Several types of molecules are offered to avoid the biological effects of IL-1β and Anakinra, an IL-1β receptor agonist, used for the treatment of systemic inflammatory disorders, has been proposed as a therapeutic strategy for PCa treatment [16].

IL-1β production is tightly controlled both at transcriptional and post-translational level. Primary signals lead to the expression of the biologically inactive intracellular precursor (pro-IL-1β) via the activation of inflammatory pathways such as nuclear factor-κB (NF-κB) and extracellular signal regulated-kinases (ERK1/2). Secondary activating signals lead to the assembly of the inflammasome platform. This event allows the activation of caspase-1, which in turn, is responsible for the proteolytic maturation of IL-1β, pivotal for its secretion [17].

Inflammasomes are cytoplasmic molecular platforms composed of receptors, such as nucleotide-binding oligomerization domain (NOD)-like receptor (NLRs) and absent in melanoma 2 (AIM2)-like receptors (ALRs), the adaptor apoptosis-associated speck like protein containing a caspase-recruitment domain (ASC), and the effector caspase-1 [17]. Upon activation, the inflammasome platform assemblies and leads to caspase-1 activation, which, in turn, is responsible for the maturation of inflammatory cytokines, including IL-1β and IL-18. Inflammasome inappropriate activation, creating a pro-inflammatory microenvironment and suppressing local immunity, appears as an emerging player in mediating cancer initiation, progression, and metastasis [18,19].

NOD-like receptor protein 3 (NLRP3) is the best characterized inflammasome receptor, and its inappropriate activation has been correlated with the development and progression of gastrointestinal, skin, breast, and hepatocellular cancer [20] and to the progression of late-stage melanoma [13]. The clinical relevance of NLRP3-inflammasome in multiple forms of cancer highlights its therapeutic promise as a molecular target [19].

In prostate tissue, pathogens and destructive signals are stimulatory factors for NLRP3-inflammasome activation [21], but its role in PCa remains uncharacterized and poorly understood.

We have recently demonstrated a powerful inhibitory action of Atrial Natriuretic Peptides (ANP) and B-Type Natriuretic Peptide (BNP) on all the routes of NLRP3-inflammasome activation in human monocytic cells via the modulation of multiple molecular pathways [22]. ANP and BNP are autocrine/paracrine/endocrine factors belonging to the family of Natriuretic Peptides (NPs). NPs are deeply involved in cardiovascular homoeostasis, mainly through blood volume and pressure regulation. Most of the function of ANP and BNP are mediated via the guanylyl cyclase Natriuretic Peptide Receptor-1 (NPR-1) expressed in many cells and tissues. NPR-1 activation induces a rapid increase in cGMP intracellular levels and the subsequent activation of cGMP-dependent phosphodiesterases, cGMP-dependent protein kinases (PKG-I and -II), cGMP-dependent ion channels [23,24]. Alongside the well-known role in cardiovascular homeostasis, NPs and NPR-1 are involved in a wide range of biological functions both in physiological and pathological conditions, including cancer [23]. ANP regulates several pathways involved in tumor development, progression, recurrence, and metastasis. In particular, ANP, by downregulating mitogen activated protein kinases (MAPK) cascade, leads to the inhibition of the proto-oncogenes c-FOS and c-JUN in cancer cells [25,26]. On these bases, ANP has been proposed as an attractive candidate for anticancer therapy [26].

The relationship between BNP and cancer-related inflammation is mainly confined to the evaluation of its plasma levels in cancer patients where its increase has been tentatively linked to cancer-related inflammation [27,28,29]. Therefore, the role of both ANP and BNP on cancer-related inflammation remains poorly investigated.

This paper aimed to analyze the contribution of both ANP and BNP in the regulation of the inflammatory behavior in prostate cancerous and non-cancerous cell lines.

We showed that advanced-stage prostate cancer PC3 cells display constitutively activated NLPR3-inflammasome that mediates the conversion of the IL-1β precursor to the secreted and active form of IL-1β. Moreover, PC3 cells express low levels of ANP and BNP compared to androgen-sensitive LNCaP cells, which do not present an active NLRP-3 inflammasome platform. The exogenous administration of ANP and BNP to PC3 cells, leading to the phosphorylation of NLRP3 receptor and by inhibiting NF-κB and p38-MAPK signaling pathway, reduce NLRP3-inflammasome platform constitutive activation. Moreover, we also showed that ANP and BNP revert the inflammatory phenotype induced by PC3-derived EVs in prostate non-cancerous PNT2 cells via ERK 1/2 and p38-MAPK inhibition.

## 2. Materials and Methods

### 2.1. Reagents

All the used reagents were analytical grade reagents. Unless otherwise stated reagents are from Merk KGaA (Darmstadt, Germany). Human ANP was solubilized in 5% acetic acid. Human BNP was acquired from Phoenix Europe GmbH (Karlsruhe, Germany) and solubilized in H_2_O. p38-MAPK inhibitor SB203580 was obtained from Santa Cruz Biotechnology, Inc. (Dallas, TX, USA), and dissolved in DMSO.

### 2.2. Cell Culture and Drug Treatments

Human androgen sensitive prostate adenocarcinoma LNCaP cells, human androgen-independent prostate cancer PC3 cells, and human non-cancerous prostatic epithelial PNT2 cells were purchased from the American Type Culture Collection (ATCC, Manassas, VA, USA). All cell lines were cultured at 37 °C in 5% CO_2_ in RPMI 1640 supplemented with 10% heat inactivated FBS, L-glutamine, 100 units/mL of penicillin, and 0.1 mg/mL of streptomycin (Invitrogen, Italy).

Cells were seeded at a density of 4.2 × 10^4^ cells/cm^2^ and incubated for 24 or 48 h before treatments or analysis. PC3 cells were treated with ANP or BNP (1 µM) for 24 h. In independent experiments, PC3 cells were pre-incubated with SB203580 (50 µM) for 1 h before treatment with ANP or BNP (1 µM) for 24 h. In independent experiments, PNT2 cells were treated with ANP or BNP (1 µM) for 10 min before exposure to PC3-derived extracellular vesicles (PC3-EVs) (100 µg/mL). DMSO (0.5%) or acetic acid (0.0005%) produced no significant toxicity. Control cells with DMSO or acetic acid did not show any significant toxicity. Whole cell lysates were obtained using RIPA buffer with protease and phosphatase inhibitors.

### 2.3. Extracellular Vesicle Isolation

Extracellular vesicles (EVs) were isolated from PC3 cells by sequential centrifugation steps as previously described [30]. EVs were resuspended in PBS with 1% penicillin/streptomycin solution. Absorbance at 280 nm was used to determine protein concentration. EVs were stored at −80 °C until use.

### 2.4. Western Blot Analysis

Total proteins (20µg) were subjected to sodium dodecyl sulfate-polyacrylamide gel electrophoresis (SDS-PAGE) and transferred to nitrocellulose membrane as previously described [8]. The membranes were blotted overnight at 4 °C with the anti-human Abs diluted 1:1000 in Roti-Block (Table 1) and incubated for 1 h at room temperature with the appropriated HRP-conjugated secondary Abs (1:2000 dilution). Bands were revealed using the enhanced chemi-luminescence (ECL) system. Membranes were stripped and re-probed with anti-β-actin or α-tubulin as loading control. Densitometric analyses were performed with ImageJ software (https://imagej.nih.gov/ij/).

### 2.5. Measurements of Secreted IL-1β

Secreted IL-1β was measured in 100 µL cell culture medium collected after 24 or 48 h culture by the specific ELISA kit, according to the manufacturer’s instruction (eBioscience, San Diego, CA, USA).

### 2.6. Quantitative Real Time PCR Analysis

Total RNA was isolated using TRIzol Reagent (Invitrogen, Milan, Italy) according to the manufacturer’s guidelines. Total RNA (1µg) was retro-transcribed using RevertAid^TM^ H Minus First Strand cDNA Synthesis Kit (Fermentas, Hanover, MD, USA) and random primers System (Invitrogen, Milan, Italy), according to the manufacturer’s instructions. Real-time PCR was performed on a Mx3000P QPCR Systems (Agilent Thecnologies, Santa Clara, CA, USA) to analyze the expression of ANP, BNP, NPR1 using GAPDH as housekeeping gene. The primer sequences (200 nM) used for RT-PCR are reported in Table 2.

The thermal cycling conditions were as follows: 1 cycle at 95 °C for 10 min, followed by 40 cycles at 95 °C for 1 min, 59 °C for 30 s and 72 °C for 30 s.

PCR reactions were performed in a total volume of 25 μL, containing 500 ng of cDNA, 1X Reagent Brilliant II SYBR Green QPCR Master Mix, 0.5 µL of ROX Reference Dye, and a concentration of specific primers. Data for comparative analysis of gene expression were obtained using the ΔΔCt method.

### 2.7. Statistical Analysis

Results were expressed as means ± SD of at least three independent experiments performed in triplicate. Statistical significance of differences was assessed by Student’s *t*-test and considered significant when *p* < 0.05.

## 3. Results

### 3.1. LNCaP and PC3 Cells Display Different Inflammatory Phenotype

Inflammatory response represents a key event in PCa development and progression. Inflammasomes are central players in inflammation and their inappropriate activation has been implicated in many forms of cancer [19].

**Figure 1:**
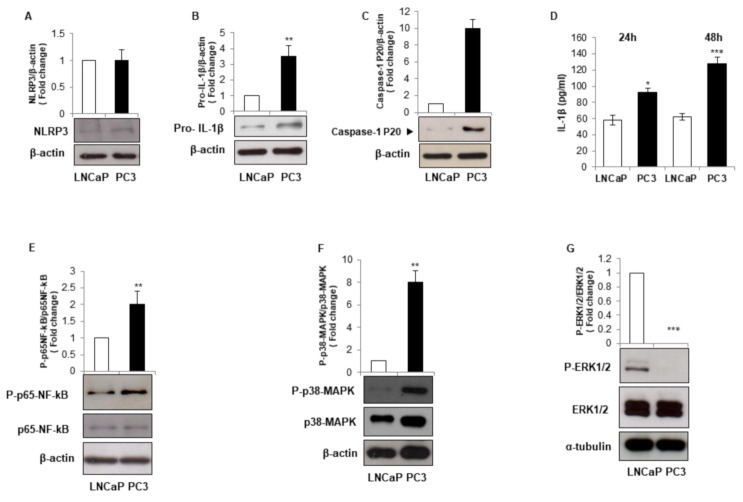
LNCaP and PC3 cells display different inflammatory phenotype. Prostate cancer LNCaP and PC3 cells were grown for 24 h, and cell lysates were immunoblotted for NLRP3 (**A**), pro- IL-1β (**B**), mature caspase-1-p20 (**C**), phospho-p65-NF-κB or p65-NF-κB (**E**), phospho-p38-MAPK or p38-MAPK (**F**), and phospho-ERK 1/2 or ERK 1/2 (**G**). β-actin or α-tubulin were used as a loading control. Representative Western blots images are shown. Histograms represent densitometric quantification. LNCaP cells were used as control and assumed as 1. All histograms indicate the mean ± SD of at least *n* = 3 independent experiments each one tested in triplicate. ** *p* < 0.01, *** *p* < 0.001 versus LNCaP cells; (**D**) prostate cancer LNCaP and PC3 cells were grown for 24 or 48 h, and the supernatants were collected for IL-1β detection by ELISA. * *p* < 0.05, *** *p* < 0.001 versus LNCaP cells.

We first asked whether LNCaP and PC3 cell lines, characterized by different androgen responsiveness and thus modelling different tumor progression stages, may have a different inflammatory profile regarding NLRP3-inflammasome activation and IL-1β maturation. We found that the advanced stage prostate cancer PC3 cells, exhibit comparable NLRP3 protein expression level but higher pro-IL-1β and active P-20 fragment of caspase-1 (Figure 1A–C), paralleled by doubled IL-1β secretion level after 48 h of culture (Figure 1D) compared to the androgen-sensitive LNCaP cells. These data indicate that PC3 cells-increased ability to secrete active IL-1β relies on constitutive inflammasome activation. Different molecular signaling pathways are involved in inflammasome activation, such as nuclear factor-κB (NF-κB), extracellular signal regulated-kinases (ERK1/2), p38 mitogen activated protein kinases (p38-MAPK) [31,32,33]. Although the total protein expression level was unchanged, we found an enhanced phosphorylation of p65-NF-κB and p38-MAPK in PC3 compared to LNCaP cells (Figure 1E,F) and a strong phosphorylation of ERK1/2 MAPK in LNCaP cells in comparison to PC3 cells (Figure 1G). These data indicate that NF-κB, ERK1/2 MAPK, and p38-MAPK may be involved in the acquisition of the different inflammatory profile that characterizes the two cell lines.

### 3.2. LNCaP and PC3 Cells Display Different Natriuretic Peptides Expression Levels

**Figure 2:**
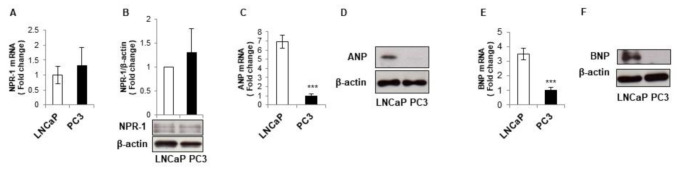
LNCaP and PC3 cells display different Natriuretic Peptides expression levels. Prostate cancer PC3 and LNCaP cells were grown for 24 h. Gene and protein expression were assessed for NPR-1 (**A**,**B**), ANP (**C**,**D**), and BNP (**E**,**F**). Gene expression was assessed by RT-PCR and values were normalized to GAPDH and presented as 2^−∆∆Ct^. Relative mRNA gene abundance in PC3 cells were assumed as 1. *** *p* < 0.001 vs. PC3 cells. Protein expression was evaluated in total extract analyzed by Western blotting. β-actin was used as a loading control. Representative Western blots images of at least *n* = 3 independent experiments, each one tested in triplicate, are shown.

We have already shown that ANP and BNP interfere with all the routes of caspase-1-inflammasome activation in monocytic cells via the modulation of multiple molecular pathways [22]. NPs capability to counteract inflammasome activation in immune cells, let us hypothesize that a different NPs and/or NPR-1 expression profile could be responsible for the dissimilar constitutive caspase-1/inflammasome activation detected in LNCaP and PC3 cells. We found that NPR-1 expression was comparable in the two cell lines at both gene and protein level (Figure 2A,B). On the other hand, LNCaP cells expressed higher amounts of ANP and BNP at both gene and protein levels compared to PC3 cells (Figure 2C–F).

### 3.3. ANP and BNP Counteract Constitutive Inflammasome Activation in PC3 Cells

**Figure 3:**
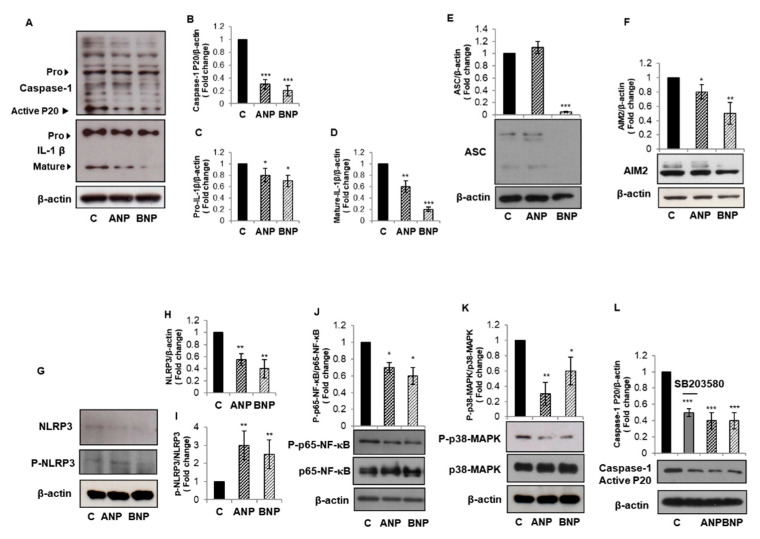
ANP and BNP counteract constitutive inflammasome activation in PC3 cells. PC3 cells were treated with ANP or BNP (1 µM) for 24 h. Cell lysates were immunoblotted for caspase-1 (**A**,**B**), IL-1β (**A**,**C**,**D**), ASC (**E**), AIM2 (**F**), phospho-NLRP3 (Ser295) or NLRP3 (**G**–**I**), phospho-p65-NF-κB or p65-NF-κB (**J**), phospho-p38-MAPK or p38-MAPK (**K**). PC3 cells were pre-incubated for 1 h with SB203580 (50 µM) and then treated with ANP or BNP (1 µM) for 24 h. Cell lysates were immunoblotted for caspase-1 (**L**). β-actin was used as a loading control. Representative Western blots images are shown. Histograms represent densitometric quantification. All histograms indicate the mean ± SD of at least *n* = 3 independent experiments, each one tested in triplicate. * *p* < 0.05, ** *p* < 0.01, *** *p* < 0.001 vs. untreated PC3 cells.

Based on the potent anti-inflammatory and immune modulatory role exerted by ANP and BNP in THP1 cells, we hypothesize that exogenous NPs could revert constitutive inflammasome activation in PC3 cells that express NPR-1 receptor. We exposed PC3 cells to 1 µM ANP or BNP for 24 h. The treatment with both NPs diminished caspase-1 activation, as evidenced by the statistically significant reduction in the p20 active/mature form (Figure 3A,B). In the same experimental conditions, ANP and BNP slightly reduced pro-IL-1β expression (Figure 3A,C), while strongly diminishing its mature form (Figure 3A,D). To gain more insight on inflammasome activation, we analyzed protein levels of other inflammasome cascade components. We found that BNP, but not ANP, diminished the protein level of the adaptor ASC (Figure 3E) and both NPs diminished the expression profile of the cytoplasmatic sensors AIM2 (Figure 3F) and NLRP3 (Figure 3G,H). We recently demonstrated that NPs, via PKG-I activation induce the phosphorylation of NLRP3 at Ser295, leading to inflammasome platform disassembly in THP1 cells [22]. To verify whether NPs can exert the same effects in prostate cancer PC3 cells, we analyzed the effect of 1 µM ANP or BNP treatment for 24 h on Ser295 phosphorylation of NLRP3 in PC3 cells. We found that both NPs raised the level of phospho-NLRP3 of almost three times (Figure 3G,I). These results suggest that NPs inhibit NLRP3-inflammasone cascade in PC3 cells by phosphorylating the NLRP3 cytoplasmatic receptor. To determine the molecular mechanism responsible for NPs-induced inhibition of inflammasome activation and IL-1β maturation in PC3 cells, we analyzed the activation of NF-κB and p38-MAPK signaling pathways, known to be linked to inflammasome activation [31,32]. We found that both NPs induced a slight reduction in NF-κB phosphorylation (Figure 3J) and a strong reduction in p38-MAPK phosphorylation (Figure 3K). In order to demonstrate the involvement of p38-MAPK pathway in inflammasome activation in PC3 cells, we used the specific p38-MAPK inhibitor SB203580. We found that a 1 h pretreatment with 50 µM SB203580 reduced caspase-1 activation to the same extent as ANP and BNP (Figure 3L). (Figure 3L). These data suggest that NPs can interfere with inflammasome activation through both NF-κB and p38 MAPK-dependent routes.

### 3.4. ANP and BNP Counteract PC3-Derived EVs-Induced Inflammasome Activation in PNT2 Cells

**Figure 4:**
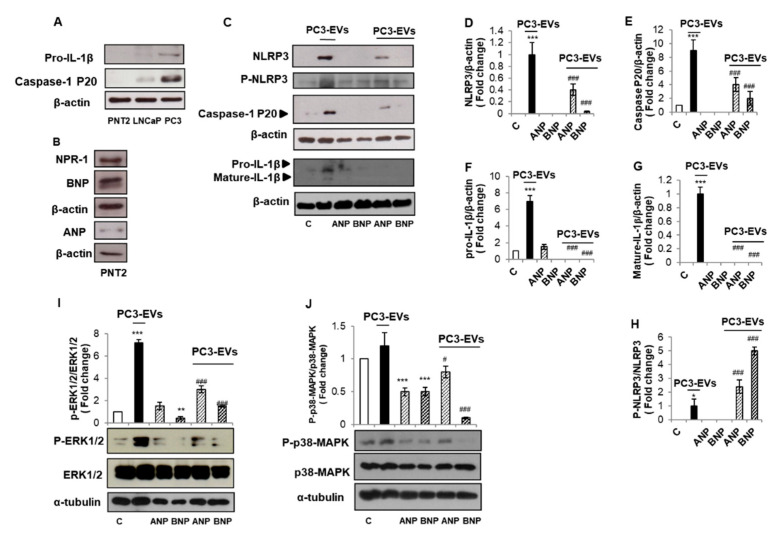
ANP and BNP counteract PC3-derived EVs-induced inflammasome activation in PNT2 cells. Non-cancerous PNT2 cells were grown for 24 h. Cell lysates were immunoblotted for pro- IL-1β and mature caspase-1-p20 (**A**); NPR-1, ANP, and BNP (**B**). PNT2 cells were pre-incubated with ANP or BNP (1 µM) for 10 min and then treated with PC3-derived EVs (PC3-EVs) (100 µg/mL) for a 24 h. Cell lysate were immunoblotted for NLRP3 (**C**,**D**), phospho-NLRP3 (Ser295) (**C**,**H**), caspase-1 (**C**,**E**), IL-1β (**C**,**F**,**G**), phospho-ERK 1/2 or ERK 1/2 (**I**) and phospho-p38-MAPK or p38-MAPK (**J**). β-actin or α-tubulin were used as a loading control. Representative Western blots images are shown. Histograms represent densitometric quantification. All histograms indicate the mean ± SD of at least *n* = 3 independent experiments, each one tested in triplicate. ** *p* < 0.01, *** *p* < 0.001 vs. untreated PC3 cells. ^#^ *p* < 0.05, ^###^ *p* < 0.001 vs. PC3-EVs treated PC3 cells.

We have demonstrated that PC3-derived EVs (PC3-EVs) may contribute to the auto-perpetuating inflammatory loop typical of tumor microenvironment by inducing inflammasome activation in non-cancerous PNT2 cells [8]. We first confirmed that pro-IL1β and mature/active caspase-1 expression is very low/absent in unstimulated PNT2 cells (Figure 4A) [8]. In order to assess whether NPs can also modify PC3-EVs-induced NLRP3-inflammasome cascade activation, we determined the basal expression of NPR-1 and NPs in PNT2 cells. We found that NPR-1, ANP, and BNP are expressed in PNT2 cells (Figure 4B). PNT2 were pre-incubated with 1 µM ANP or BNP for 10 min before the exposure to 100 µg/mL of PC3-EVs for 24 h. We found that ANP reduced and BNP abrogated PC3-EVs-induced NLRP3 expression and caspase-1 activation (Figure 4C–E) and both NPs abrogated pro- and mature-IL-1β expression (Figure 4C,F,G), indicating that NPs can also counteract inflammatory signals provided by exogenous factors. In addition, we found that both NPs, although to a different extent, act through NLRP3 Ser295 phosphorylation (Figure 4C,H). We previously demonstrated that the molecular mechanism at the basis of PC3-EVs-induced inflammasome activation in PNT2 cells involves ERK1/2 [8], a mitogen activated protein kinase already connected to the inflammasome activation [32]. To analyze whether NPs could counteract PC3-EVs-induced activation of ERK 1/2, we pre-incubated PNT2 cells with 1 µM ANP or BNP for 10 min before the exposure to 100 µg/mL of PC3-EVs for 24 h. We found that ANP strongly reduced, and BNP completely abrogated the consistent activation of ERK1/2 induced by PC3-EVs (Figure 4I). Due to the observed inhibitory effect of ANP and BNP on p38 MAPK in PC3 cells (Figure 3), we wondered whether a similar effect could be exploited also in PC3-EVs-exposed PNT2 cells. We found that although PC3-EVs treatment does not induce a significant modification in the phosphorylation level of p38-MAPK (Figure 4J), both NPs strongly inhibited p38MAPK phosphorylation in PNT2 cells in all the analyzed experimental conditions.

These results point out that ANP and BNP counteract PC3-EVS-induced inflammatory behavior on non-cancerous cells and suggest that NPs may contribute to balancing the tumor inflammatory microenvironment.

## 4. Discussion

In this study, we demonstrated that the Natriuretic Peptides (NPs): atrial natriuretic peptide (ANP) and B-type natriuretic peptide (BNP), through NLRP3 phosphorylation, inhibit constitutive- and extracellular vesicles (EVs)-induced inflammasome activation via different signaling pathways in prostate cell lines.

Inflammasomes are cytoplasmic molecular platforms composed of receptors, the adaptor protein ASC, and the effector caspase-1 [17]. Upon activation of different pathways, such as nuclear factor-κB (NF-κB), extracellular signal regulated-kinases (ERK1/2), p38 mitogen activated protein kinases (p38 MAPK) [31,32,33], the inflammasome platform assembles, and leads to caspase-1 activation. This latter, in turn, is responsible for the maturation of inflammatory cytokines, including IL-1β and IL-18 [17].

Although the pivotal role for inflammasome in cancer has been thoroughly demonstrated, the role of inflammasomes in prostate cancer is still to be clarified. It has been shown that the inflammasome receptor NLRP3 recognizes a diverse set of pro-inflammatory stimuli including metabolites that are actively secreted in an extracellular microenvironment during cancer expansion. These include ATP, which can activate NLRP3 inflammasome via the purinergic P2 × 7 receptors and, specifically in prostate tissue, uric acid, crystals, and infections, which have all been involved in prostate cancer progression [19].

The complexity of tumor microenvironment is further increased by the presence of EVs, membrane-enclosed spherical particles, which are devoted to the transport of active biomolecules between cells, thus guaranteeing an active intercellular communication [6].

It has been exhaustively demonstrated that the crosstalk between tumor microenvironment and local inflammation promotes oncogenic initiation, development, and progression. In this context, inflammasome seem to play a crucial role. Its activation has been indeed connected to all the hallmarks of cancer [19]. For example, NLRP3 inflammasome activation in tumor cells supports a chronic inflammatory microenvironment, which promotes malignant transformation and suppress local immunity [19]. NLRP3 activation in cancer cells and the consequent IL-1β secretion have been associated with transmitting signals that stimulate normal cells within the tumor microenvironment and supply them with growth factors [34]. We recently demonstrated that EVs secreted by prostate cancer PC3 cell line (PC3-EVs) could induce NLRP3 inflammasome activation in non-cancerous PNT2 prostate cells, thus supporting the tumor-promoting microenvironment [8]. These results indicate that crosstalk between cancer cells and other tumor microenvironment residing cells is bidirectional and can provide cooperative signals that favor tumor progression. On this basis, inflammasome inhibition seems to be a promising therapeutic strategy for cancer treatment.

In this light, a great importance is assumed by biomolecules for their synthesis easiness, low toxicity, and high target specificity and selectivity [35].

The biomolecules ANP and BNP are endogenous peptides secreted from the heart and signaling, through NPR-1, to reduce cardiac hypertrophy and ventricular fibrosis [24]. A role for NPs in inflammation has also been described. It has been demonstrated that inflammation increases the expression of NPs [36] and that NPR-1 is implicated in immune and inflammatory reactions [37]. ANP and BNP secretion is modulated by the inflammatory cytokines interleukin (IL)-1, IL-6, and tumor necrosis factor (TNF)-α [38,39]; ANP and BNP act by reducing inflammasome activation in monocytic cells [22,31,32].

In this paper we showed that ANP and BNP pre-treatment of non-cancerous prostate PNT2 cells could reduce inflammasome activation induced by PC3-EVs. In fact, both the NPs decrease the expression and increase Ser 295 phosphorylation of NLRP3 and strongly reduce the mature/active form of caspase-1 and mature IL-1β. These results suggest that ANP and BNP counteract the pro-inflammatory action of EVs secreted by cancer cells, thus potentially dampening inflammation in the tumor microenvironment. The contribution of inflammasome activation in the generation of a tumor-supporting microenvironment is underpinned by the finding that NLRP3 activation in breast cancer-associated fibroblasts modify the expression of adhesion molecules, thus promoting metastatic spread [11]. In agreement, NLRP3 knock-out mice, orthotopically transplanted with breast cancer cells, display less pulmonary metastases [40]. Moreover, the crosstalk between colorectal carcinoma cells and microenvironment-infiltrating immune cells induces NLRP3 inflammasome activation in macrophages and the blockage of NLRP3 signaling by NLRP3 antagonist, glibenclamide or caspase-1 antagonist, AC-YVAD-cmk, suppressed colorectal cancer cell migration, and liver metastasis [12].

A low constitutive expression of inflammasome components and a tight regulation of inflammasome activation are pivotal to avoid uncontrolled inflammation [41]. In this paper we showed that, compared to LNCaP cells, advanced-stage prostate cancer PC3 cells display constitutively high levels of active caspase-1 and actively secrete IL-1β. Moreover, we found that NF-κB phosphorylated/active form, which drives inflammasome components expression, was increased in PC3 cells. This result implies that molecular changes associated with tumor progression can enable advanced stage prostate cancer cells to acquire the ability to activate the inflammasome cascade. Among the molecular mechanisms regulating inflammasome cascade activation are epigenetic modification and miRNAs [41]. In particular, miRNA let-7b has been involved in the regulation of Toll-like receptor 4 (TLR4) expression, which, in turn, is responsible for NF-κB activation [42]. It has recently been shown that miRNA let-7b expression is reduced in prostate cancer PC3 cells [43], suggesting that the inhibitory mechanisms leading to the homeostatic control of NF-κB activation are dysregulated in advanced stage prostate cancer cells. Our results are in agreement with several studies demonstrating that NF-κB promotes cell survival, proliferation, and invasion in prostate cancer [5]. Because of the association between NF-κB p65 nuclear frequency and more aggressive prostate cancer, targeting NF-κB has been suggested as a promising therapeutic option [5].

The stimulus-independent inflammasome activation observed in PC3 cells can further contribute to the establishment of the pro-inflammatory, tumor-promoting microenvironment. The analyses of constitutive inflammasome activation in LNCaP and PC3 cell revealed that LNCaP cells possess very low/absent levels of inflammasome activation. For this reason, we choose PC3 cells to investigate NPs’ ability to reduce basal inflammasome activation. We showed that both NPs could counteract constituent inflammasome activation in PC3 cells. We found that pre-treatment with ANP and BNP slightly reduced NF-κB phosphorylation/activation and NLRP3 expression. However, NPs strongly diminished the mature/active form of caspase-1 and IL-1β. We have recently demonstrated that ANP and BNP, via PKG-I activation, induce the phosphorylation of NLRP3 at the Ser 295 position, thus impeding the assembly of the inflammasome platform in THP-1 cells [21]. Here, by showing that pre-treatment with ANP and BNP increases the levels of phosphorylated NLRP3, we depict the intracellular mechanism that leads to the inhibition of constitutively active NLRP3 in PC3 cells.

We also showed that PC3 cells, although expressing the NPs receptor NPR-1, express neither ANP nor BNP, suggesting that advanced stage prostate cancer cells cannot experience an autocrine NPs-mediate signaling for the control of inflammasome activation. The importance of NPs signaling in prostate cancer is also supported by the finding that the expression of natriuretic peptide clearance receptor, whose main function is to remove NPs from the bloodstream [24], is increased in prostate cancer [44].

We have found that NPs-related inflammasome inhibition is mediated by the attenuation of MAPK signaling. The involvement of ERK1/2 MAPK in the mechanism underpinning ANP- and BNP-mediated inflammasome inhibition was already demonstrated [22,31,32]. Here we showed that both NPs attenuated EVs-induced ERK1/2 phosphorylation in PNT2 cells. This result is in line with the finding that ANP and long-acting natriuretic peptide show anticancer effects in human prostate adenocarcinoma by inhibiting Ras MAPK signaling pathway [45].

In our experimental conditions, although ERK1/2 phosphorylation was undetectable in PC3 cells, NPs can still inhibit inflammasome activation. Notwithstanding, PC3 cells exhibit constitutive high levels of phosphorylated p38MAPK. Several reports in the literature demonstrate that NPs inhibit p38-MAPK activation [45,46,47] and p38-MAPK activation was associated with inflammasome activation [48,49]. Similarly, several studies established the importance of the activation of p38-MAPK signaling and the anti-apoptotic, pro-survival, pro-inflammatory, and pro-angiogenic events, as well as EMT activation and invasion in PCa cell lines [50,51]. We showed that pre-treatment of PC3 cells with ANP and BNP dampen basal p38-MAPK activation, and p38-MAPK inhibition by the specific inhibitor mimics NPs-induced inflammasome inhibition. These results suggest that p38-MAPK is involved in the molecular mechanism underlying NPs-induced inflammasome inhibition.

## 5. Conclusions

In conclusion, we have shown that ANP and BNP are capable of inhibiting inflammasome activation independently from the cell type or the activating stimuli. It is to strengthen that, depending on the molecular signature of the cell, ANP and BNP can affect different signaling pathways, which, however, culminate in the inhibition of the inflammasome cascade. Furthermore, the effects of NPs are completely independent from the inflammasome activating stimuli, indicating that they can potentially exert a pleiotropic effect. It should also be underlined that, in our experimental conditions, both ANP and BNP are capable of exerting inhibitory effects on the inflammasome cascade. Several reports claim an antitumoral action for ANP. However few reports, if any, propose BNP as a therapeutic option for cancer. Our results point instead to the potential use of both NPs as therapeutic options to counteract inflammasome activation in cancer. The value of ANP for cancer therapy is also strengthened by the fact that neither cytotoxicity nor single-side effects is associated with its administration. In fact, ANP is already in clinical use for the treatment of heart failure [52]. Moreover, the availability of synthetic ANP (Anaritide or Carperitide) and BNP (Nesiritide) which has already been studied for heart failure treatment [53], let us to hypothesize NPs as novel repurposable biomolecular candidates for prostate cancer with a potential rapid bench to bedside transition.

## Figures and Tables

**Table 1:** List of Antibodies.

Target Protein (Clone)	Host	Code #	Manufacturer
mAb anti-NLRP3 (D4D8T)	Rabbit	15101T	Cell Signalling Technology
mAb anti-AIM2(D5 × 7K)	Rabbit	12948T	Cell Signalling Technology
mAb anti-ASC/TMS1 (E13EI)	Rabbit	13833T	Cell Signalling Technology
mAb anti-Phospho-NF-κB p65 (Ser536) (93H1)	Rabbit	3033	Cell Signalling Technology
mAb anti-IL1-β (3A6)	Mouse	12242	Cell Signalling Technology
mAb anti-phospho-p38 MAPK (Tyr180/Tyr182) (D3F9)	Rabbit	4511	Cell Signalling Technology
mAb anti-p38 MAPK (D13E1)	Rabbit	8690T	Cell Signalling Technology
pAb anti-Caspase-1	Rabbit	2225	Cell Signalling Technology
pAb anti-Phospho-p44/42 MAPK (ERK1/2) (Thr202/Tyr204)	Rabbit	9101	Cell Signalling Technology
anti-p44/42 MAPK (ERK1/2)	Rabbit	9102	Cell Signalling Technology
pAb anti-NF-κB p65	Rabbit	3034	Cell Signalling Technology
pAb anti-ANP	Rabbit	PA5-29559	Thermo Fisher Scientific
pAb anti-BNP	Rabbit	PA5-21321	Thermo Fisher Scientific
pAb anti-NPR-1	Rabbit	PA5-29049	Thermo Fisher Scientific
pAb anti-phospho-NLRP3 (Ser 295)	Rabbit	PA5-105071	Thermo Fisher Scientific
pAb anti-β-actin	Rabbit	4967	Cell Signalling Technology
mAb α-Tubulin (DM1A)	Mouse	3873	Cell Signalling Technology

**Table 2:** List of primers.

Gene Name	Gene Symbol	Primer Sequences (F: Forward R: Reverse)
Atrial Natriuretic Peptide	ANP	F: TCAGCCCAGCCCAGAGAG R: GCTCCAATCCTGTCCATCCTG
Brain Natriuretic Peptide	BNP	F: GAGGGCAGGTGGGAAGCAAAC R: GCAAGAAGAGCAGGAGCAGGAG
Natriuretic Peptide Receptor 1	NPR-1	F: TAACACGCACGCACACTC R: CTATGGGAAGGAGCAGGAG
Glyceraldehyde-3-phosphate dehydrogenase	GAPDH	F: TGGTATCGTGGAAGGACTCATGAC R: ATGCCAGTGAGCTTCCCGTTCAGC

## Data Availability

Data are contained within the article.
